# Primary Malignant Melanoma of the Genitourinary Tract with Upper and Lower Tracts Involvement

**DOI:** 10.1155/2013/217254

**Published:** 2013-08-28

**Authors:** Broderick Sutton, Robert Chan, Mark Sutton, Timothy Boone

**Affiliations:** ^1^Department of Urology, The Methodist Hospital, Houston, TX 77030, USA; ^2^Scott Department of Urology, Baylor College of Medicine, Houston, TX 77030, USA

## Abstract

A 91-year-old female presented with lower extremity swelling and shortness of breath. Laboratory analysis revealed elevations in blood urea nitrogen and creatinine along with microscopic hematuria on urinalysis. Computed tomography imaging showed moderate right hydronephrosis with dilatation of the proximal ureter with a soft tissue density at a transition point. Endoscopic evaluation revealed multiple raised, fleshy, and hemorrhagic masses throughout the bladder which are present in both ureters. Biopsy of these lesions revealed malignant melanoma invading the lamina propria. No dermatologic lesions were identified suggesting a primary malignant melanoma of the genitourinary system.

## 1. Introduction

Primary malignant melanoma of the bladder is a very rare lesion of the genitourinary tract accounting for only 0.2% of all patients with melanoma. Melanoma of the bladder is most commonly secondary to metastatic spread from a primary lesion originating from the skin. In the literature, only 20 prior cases of malignant melanoma of the bladder have been reported to date [1, 2]. The diagnosis of primary genitourinary melanoma requires exclusion of other melanoma sites.

Ureteral malignant melanoma is even more rarely seen and usually results from metastatic spread [3]. We present a case of primary malignant melanoma of the genitourinary tracts with concomitant upper and lower tract involvement.

## 2. Case Presentation

A 91-year-old African American female with a history of diabetes, chronic renal insufficiency, and congestive heart failure presented with bilateral lower extremity swelling and shortness of breath. She had no prior history of smoking. Her physical exam was significant for bilateral lower extremity 2+ pitting edema to midthigh. Lab work demonstrated an elevated creatinine of  1.45 with microscopic hematuria on urinalysis. 

Ultrasonography demonstrated moderate right hydronephrosis. A noncontrast CT scan of the abdomen and pelvis showed moderate right hydronephrosis with dilatation of the proximal right ureter and a soft tissue density at the transition point.

**Figure 1:**
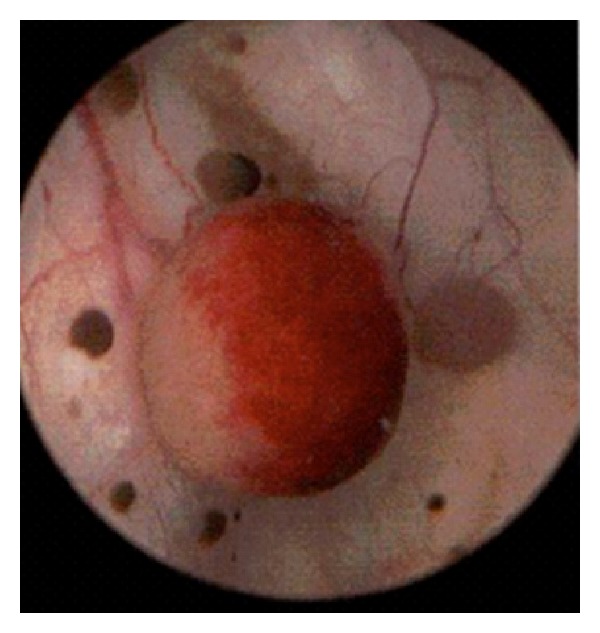
Cystoscopy demonstrating several malignant melanoma lesions throughout the bladder.

**Figure 2:**
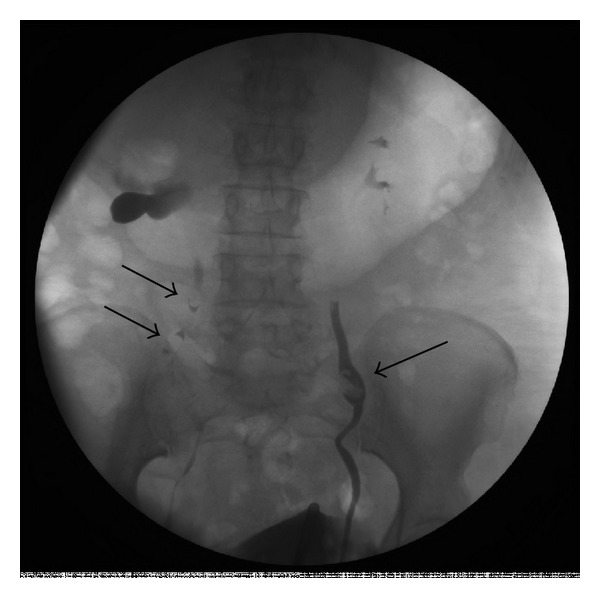
Retrograde pyelograms demonstrating bilateral filling defects in both ureters (black arrows).

She underwent endoscopic evaluation in the operating room. Cystoscopy demonstrated multiple variable-sized lesions scattered throughout the entire bladder as seen in Figure 1. These were characterized to be fleshy and raised with some noted to be hemorrhagic. Bilateral retrograde pyelograms demonstrated multiple filling defects throughout both ureters as seen in Figure 2. There were multiple filling defects in the right ureter and an ovoid midureteral filling defect in the left ureter. Right ureteroscopy was performed and revealed multiple tumors similar in appearance to those present in the bladder. Pathologic examination of the bladder lesions revealed malignant melanoma invasive into the lamina propria with stains strongly positive for S100 and melanoma cocktail. After operation, a thorough dermatologic exam was performed, however, no lesions were identified. 

The diagnosis of primary malignant melanoma of the genitourinary system with concomitant upper and lower tracts involvement was made.

After extensive discussion with the patient and her family concerning treatment options, they elected for observation. She expired within one year.

## 3. Discussion

Primary malignant melanoma of the urinary tract is extremely rare. Usually melanoma present in the urinary tract emerges as a metastatic focus from a primary dermatologic lesion. Clinically, ureteral involvement can present with symptoms of renal colic with hydronephrosis, hydroureteronephrosis, or filling defects seen on abdominal imaging. Bladder involvement can present with similar lower urinary tract symptoms as other benign entities such as benign prostatic hyperplasia or overactive bladder. These symptoms include dysuria, urgency, frequency, and nocturia. Similar to other types of bladder carcinomas, malignant melanoma of bladder will initially present with hematuria. This indicates locally advanced disease [4]. 

Definitive diagnosis includes endoscopic evaluation and biopsy with histopathological examination of the biopsy specimens. 

Treatment options for a primary bladder malignant melanoma include transurethral resection, partial or radical cystectomy, radiotherapy, immunotherapy, and chemotherapy.

 For localized disease, cystectomy is the preferred treatment option. Immunotherapy with interferon alpha or radiation therapy can also be employed if the lesions are unresectable [2]. Regardless of treatment modality, the prognosis is poor and none of the patients survived more than three years following the procedure [1, 5–7]. 

For ureteral malignant melanoma, Gakis et al. had proposed a therapeutic treatment algorithm [8]. If the lesions were unresectable, then radiation therapy and chemotherapy were treatment options. For unilateral involvement, a nephroureterectomy with regional lymphadenectomy was suggested. For bilateral involvement, partial ureterectomy and bilateral lymphadenectomy were recommended. Adjuvant chemotherapy with dacarbazine was indicated if the pathology results demonstrated positive margins or positive lymph nodes. 

## 4. Conclusion

We reported a rare case of primary malignant melanoma of the genitourinary tract involving the bladder and both ureters. A review of the different treatment options was presented; however, the prognosis of this entity is uniformly poor regardless of treatment modality.
